# Development of an efficient search filter to retrieve systematic reviews from PubMed

**DOI:** 10.5195/jmla.2021.1223

**Published:** 2021-10-01

**Authors:** José Antonio Salvador-Oliván, Gonzalo Marco-Cuenca, Rosario Arquero-Avilés

**Affiliations:** 1 jaso@unizar.es, Professor, School of Medicine, Department of Library and Information Science, University of Zaragoza, Spain; 2 gmarco@unizar.es, Professor, School of Medicine, Department of Library and Information Science, University of Zaragoza, Spain; 3 carquero@ucm.es, Professor, Department of Library and Information Science, Complutense University of Madrid, Madrid, Spain

**Keywords:** search filter, systematic reviews, PubMed, information retrieval, search strategies

## Abstract

**Objective:**

Locating systematic reviews is essential for clinicians and researchers when creating or updating reviews and for decision-making in health care. This study aimed to develop a search filter for retrieving systematic reviews that improves upon the performance of the PubMed systematic review search filter.

**Methods:**

Search terms were identified from abstracts of reviews published in *Cochrane Database of Systematic Reviews* and the titles of articles indexed as systematic reviews in PubMed. Both the precision of the candidate terms and the number of systematic reviews retrieved from PubMed were evaluated after excluding the subset of articles retrieved by the PubMed systematic review filter. Terms that achieved a precision greater than 70% and relevant publication types indexed with MeSH terms were included in the filter search strategy.

**Results:**

The search strategy used in our filter added specific terms not included in PubMed's systematic review filter and achieved a 61.3% increase in the number of retrieved articles that are potential systematic reviews. Moreover, it achieved an average precision that is likely greater than 80%.

**Conclusions:**

The developed search filter will enable users to identify more systematic reviews from PubMed than the PubMed systematic review filter with high precision.

## INTRODUCTION

Systematic reviews are an important source of evidence for researchers, health professionals, and health policymakers. Therefore, locating these reviews is of great interest to clinicians who must make evidence-based decisions concerning patient care and to researchers who must conduct systematic reviews to avoid duplicating existing quality research or as a prerequisite to developing a review synthesis.

Although clinicians and researchers should be able to find systematic reviews reliably and quickly [1], identifying them in databases presents difficulties, such as a lack of time and know-how of where to look and how to design a search strategy [2]. Search filters have been developed to help overcome these problems.

Search filters, also known as hedges, are combinations of terms used to retrieve information about a specific topic, research method, or study design. Search filters are designed to increase search efficiency by providing the searcher with a previously defined solution [3, 4]. They can be used for years as long as they remain effective, and they are useful not only for librarians and information professionals but also for clinicians who are not search experts and must find useful information for their practice [5].

To identify filters, searchers and researchers use resources such as InterTASC Information Specialists' Sub-Group (ISSG) Search Filter Resource and the McMaster Hedges Team filters [6–9]. These resources describe different types of filters, some of which are specialized for searching in different databases (e.g., MEDLINE, Embase, CINAHL) and platforms (e.g., Ovid and PubMed) [4, 5, 10–18].

Among the weaknesses of some existing search filters for systematic reviews is that they were developed several years ago, before evidence synthesis methods experienced a significant growth in the literature with the appearance of new types of reviews with systematic approaches and new terminology (e.g., the PubMed Systematic Reviews (SR) filter retrieves 6,905 items published in 2011 versus 31,924 items published in 2020). Also, these filters were evaluated using gold standard article sets created through hand-searching medical literature, not in large databases such as MEDLINE. More recently, there is a lack of filters for identifying articles on knowledge synthesis methodologies [19].

Some studies of systematic reviews did not apply specific filters but instead only included the terms “systematic review” or “meta-analysis” to the search strategy [15, 20–25]. This approach limits article retrieval because some systematic reviews do not contain these terms in the topic search fields (e.g., title, abstract, or descriptors) [26], use other terms, or are not identified as such anywhere in the text [27].

The PubMed SR filter was initially developed by Shojania and Bero [16]. Although it has been updated over the years, it retrieves a large number of studies that are not systematic reviews, causing a loss of precision [28–29]. This filter has also been adapted for other platforms (Ovid, EBSCO) and databases (EMBASE) [30].

Searching for systematic reviews in MEDLINE is not without issues. As of December 2018, there was no MeSH term for identifying systematic reviews as a specific publication type. These articles were indexed as a review publication type ([pt]), a category that also includes narrative reviews [31, 32], most of which are not systematic reviews [13]. In 2019, two important changes were made to the PubMed SR filter [33]: 1) the MeSH term “systematic review” as publication type was added, and 2) the search strategy was modified to improve its precision by eliminating ambiguous terms and searching for terms only in the title field. Also, meta-analysis was added as a publication type and a MeSH Heading, but not all systematic reviews are meta-analyses, and not all meta-analyses are indexed under this term [12].

The addition of systematic review as a publication type does not solve the problem of identifying systematic reviews, as many such reviews are not indexed with this term. There are two reasons for this issue: 1) articles added to PubMed are not immediately indexed with this term, and 2) human errors occur in the indexing process because authors employ many terms and synonyms to describe systematic reviews [12]. Thus, articles that are not systematic reviews are incorrectly indexed with this MeSH term, and articles that are systematic reviews are not indexed with this term.

The objective of this study was to develop a search filter for identifying systematic reviews in PubMed and to compare its performance with the PubMed SR filter, with the aim of achieving a higher recall rate with a high level of precision. In this study, the word “recall” will be used instead of “sensitivity,” as the former is the term most frequently used in the information retrieval field.

## METHOD

### Definition of a systematic review

A systematic review has two essential components: a literature search and a systematic approach that ensures the transparency and reproducibility of the review process. [34]. According to the PRISMA Statement, the search process must be explicitly reported in the methods section of a systematic review [35] and is the primary way of locating appropriate studies that will constitute the evidence base [36, 37]. These are the key requirements for the review to be systematic and to inform the reader that the search has been performed systematically.

The definition of a systematic review used in this study is based on the above and on the PRISMA-P definition [38]: any article that explicitly reports a search strategy to identify studies that meet eligibility criteria and conducts a quantitative or qualitative synthesis of the results. Although this definition does not include a critical appraisal of the evidence, it is more precise than those described in other studies, in which the use of a search strategy was the only criterion for classifying articles as systematic reviews [1, 16, 20].

We used this definition for two reasons: 1) strictly speaking, a review can be considered systematic when at least one step (e.g., search, selection, data extraction) is carried out systematically [26]; and 2) the search strategy used in the PubMed SR filter [33] retrieves systematic reviews as well as other types of reviews that may or may not be conducted systematically (e.g., literature reviews, scoping reviews, narrative reviews, qualitative reviews, evidence reviews, quantitative reviews, meta-reviews, critical reviews, mixed studies reviews, mapping reviews, Cochrane reviews, integrative reviews).

### Search term selection

Candidate terms for the filter were selected from titles and abstracts of systematic reviews using two approaches.

#### Terms extracted from abstracts of Cochrane reviews

Cochrane reviews are considered the gold standard of rigorous systematic reviews [39] and do not typically have terms in the titles that identify them as such. For example, Table 1 shows that 99.87% (14,440 (set #8)/14,459 (set #7)) of the reviews published in *Cochrane Database of Systematic Reviews* do not contain phrases with the terms “systematic” and “review” in the title field. Therefore, terms from Cochrane review abstracts were deemed suitable for distinguishing systematic reviews from other types of articles.

**Table 1:** Search strategy to retrieve Cochrane reviews from PubMed (Date of search: November 26, 2020)

Search	Query	Items retrieved	Items retrieved from set 4	Recall (base=set 4)
#1	Cochrane database syst rev [ta]	15,095		
#2	LETTER [PT] OR EDITORIAL [PT] OR COMMENT [PT] OR CASE REPORTS [PT] OR HISTORICAL ARTICLE [PT] OR REPORT [TI] OR (PROTOCOL [TI] OR PROTOCOLS [TI]) OR WITHDRAWN [TI] OR RETRACTION OF PUBLICATION [PT] OR RETRACTION OF PUBLICATION AS TOPIC [MESH] OR RETRACTED PUBLICATION [PT] OR REPLY [TI] OR PUBLISHED ERRATUM [PT]	4,582,001		
#3	#1 not #2	14,473		
#4	#3 and hasabstract	14,459		
#5	(search* [tiab] OR medline [tiab] OR pubmed [tiab] OR embase [tiab] OR Cochrane [tiab] OR scopus [tiab] OR web of science [tiab] OR sources of information [tiab] OR data sources [tiab] OR following databases [tiab]) AND (selection criteria [tiab] OR study selection [tiab] OR eligibility criteria [tiab] OR inclusion criteria [tiab] OR exclusion criteria [tiab])	83,165	14,302	98.9%
#6	#4 not #5	157		
#7	systematic review [ti] OR systematic literature review [ti] OR systematic scoping review [ti] OR systematic narrative review [ti] OR systematic qualitative review [ti] OR systematic evidence review [ti] OR systematic quantitative review [ti] OR systematic meta-review [ti] OR systematic critical review [ti] OR systematic mixed studies review [ti] OR systematic mapping review [ti] OR systematic cochrane review [ti] OR systematic search and review [ti] OR systematic integrative review [ti]	138,869	19	0.13%
#8	#4 not #7	14,440		
#9	Systematic review [pt]	139,861	14,155	97.9%
#10	#4 not #9	304		

An article set was created in PubMed consisting of all article types published in *Cochrane Database of Systematic Reviews* (Table 1, set #1). All articles that were not systematic reviews (e.g., letters, editorials, retractions, protocols) or did not have an abstract were excluded from this set.

The abstracts of the resulting 304 articles were examined, and terms related to two conceptual components (i.e., search methods and eligibility criteria) implicit in the systematic review definition used in this study were selected. Other terms found in the PRISMA Statement [35] were also included, such as the most frequently used and recommended databases [40–43], some generic terms (e.g., “databases,” “sources of information”), and terms related to study eligibility criteria. The resulting search strategy was:

(search* [tiab] OR medline [tiab] OR pubmed [tiab] OR embase [tiab] OR Cochrane [tiab] OR scopus [tiab] or web of science [tiab] OR sources of information [tiab] OR data sources [tiab] OR following databases [tiab])

AND

(selection criteria [tiab] OR study selection [tiab] OR eligibility criteria [tiab] OR inclusion criteria [tiab] OR exclusion criteria [tiab])

#### Terms extracted from the titles of articles indexed as systematic review [pt] and differing from those already in the PubMed SR filter.

For this step, a PubMed search was carried out excluding articles with PubMed SR filter terms in the title field (Table 2, set #2) as well as certain publication types (set #3). A total of 34,106 articles were retrieved, all of which were indexed as “systematic review [pt].”

**Table 2:** Search strategy to create a set of articles indexed as systematic reviews without the PubMed SR filter terms in the title field (Date of search: November 26, 2020)

Search	Query	Items
#1	systematic review [pt]	139,586
#2	systematic review* [ti] or meta-analysis [ti] or metaanalysis [ti] or systematic literature review [ti] OR systematic scoping review [ti] OR systematic narrative review [ti] OR systematic qualitative review [ti] OR systematic evidence review [ti] OR systematic quantitative review [ti] OR systematic meta-review [ti] OR systematic critical review [ti] OR systematic mixed studies review [ti] OR systematic mapping review [ti] OR systematic cochrane review [ti] OR systematic search and review [ti] OR systematic integrative review [ti]	205,047
#3	LETTER [PT] OR EDITORIAL [PT] OR COMMENT [PT] OR CASE REPORTS [PT] OR HISTORICAL ARTICLE [PT] OR REPORT [TI] OR (PROTOCOL [TI] OR PROTOCOLS [TI]) OR WITHDRAWN [TI] OR RETRACTION OF PUBLICATION [PT] OR RETRACTION OF PUBLICATION AS TOPIC [MESH] OR RETRACTED PUBLICATION [PT] OR REPLY [TI] OR PUBLISHED ERRATUM [PT]	4,582,001
#4	#1 not #2 not #3	**34,106**

The titles of these articles were screened, and an initial list of 700 different combinations of potentially candidate search terms was created (Appendix 1). Candidate terms were those that had a semantic relationship with the previously proposed definition of a systematic review. Subsequently, only terms and phrases not included in the PubMed SR filter were selected (Appendix 2).

These terms were employed to develop several search strategies organized into conceptual categories (Table 3, C1–C6, set #3 to #17). Bibliographic data for the publications retrieved by each search strategy were downloaded in a comma-separated value (CSV) data format and imported into Microsoft Excel. Other data were then recorded, such as whether the articles were systematic reviews and, in some cases, their publication type and/or terms appearing in the title field.

**Table 3:** Evaluation of search strategies to identify systematic reviews in PubMed (Date of search: November 26, 2020)

Search	Query	Items	New SR	Precision
#1	systematic [sb]	172,645		
#2	LETTER [PT] OR EDITORIAL [PT] OR COMMENT [PT] OR CASE REPORTS [PT] OR HISTORICAL ARTICLE [PT] OR REPORT [TI] OR PROTOCOL [TI] OR PROTOCOLS [TI] OR WITHDRAWN [TI] OR RETRACTION OF PUBLICATION [PT] OR RETRACTION OF PUBLICATION AS TOPIC [MESH] OR RETRACTED PUBLICATION [PT] OR REPLY [TI] OR PUBLISHED ERRATUM [PT]	4,582,001		
**C1-SYSTEMATIC REVIEWS AND OTHER TYPES**				
#3	(systematic* [ti] AND review [ti]) NOT #2 NOT #1	2886	2386	82.7%
#4	Systematic overview* [ti] NOT #2 NOT #1	191	146	76.5%
#5	Cochrane review* [ti] NOT #2 NOT #1	649	(383)+86	72.3%
#6	systemic review* [ti] NOT #2 NOT #1	538	472	87.7%
#7	scoping review [ti] OR scoping literature review [ti] OR mapping review [ti] NOT #2 NOT #1	4,458	4,302	96.5%
#8	Umbrella review* [ti] NOT #2 NOT #1	273	264	96.7%
#9	(review of reviews [ti] OR overview of reviews [ti]) NOT #2 NOT #1	145	113	77.9%
#10	(integrative review [ti] OR integrated review [ti] OR integrative overview [ti] OR meta-review [ti] OR meta-synthesis [ti] OR metasynthesis [ti] OR quantitative review [ti] OR quantitative synthesis [ti] OR research synthesis [ti] OR meta-ethnography [ti]) NOT #2 NOT #1	3,527	2,994	84.9%
#11	Systematic synthesis [ti] NOT #2 NOT #1	65	10	15.4%
**C2-SYSTEMATIC SEARCH**				
#12	Systematic literature search [ti] NOT #2 NOT #1	53	39	73.5%
#13	Systematic search [ti] NOT #2 NOT #1	210	44	20.9%
**C3-SYSTEMATIC LITERATURE RESEARCH**				
#14	Systematic literature research [ti] NOT #2 NOT #1	10	9	90.0%
**C4-SYSTEMATIC APPRAISAL OF LITERATURE OR EVIDENCE**				
#15	(systematic appraisal [ti] OR systematic assessment [ti] OR systematic evaluation [ti] OR systematic analysis [ti]) AND (literature [ti] OR evidence [ti] OR research [ti] OR studies [ti] OR trials [ti]) NOT #2 NOT #1	236	112	47.5%
**C5-ISSUES RELATED TO EVIDENCE**				
#16	(evidence based approach [ti] OR evidence based management [ti] OR evidence based treatment* [ti] OR evidence based recommendation* [ti] OR scientific evidence [ti] OR ((review* [ti] OR overview* [ti] OR synthes* [ti] OR update [ti] OR critical appraisal [ti] OR critical evaluation [ti]) AND evidence [ti])) NOT #2 NOT #1	13,763	4,541	33.0%
**C6-METAANALYSIS**				
#17	(meta-analyses [ti] OR metaanalyses [ti] OR metaanalysis [ti] OR meta-analysis [ti] OR meta-analytic review [ti] OR meta-analytical review [ti] or meta-analysis [pt]) NOT #2 NOT #1	76,522	64,894	84.8%
**DATABASES SEARCH AND ELIGIBILITY CRITERIA (Terms of abstracts)**				
#18	(search* [tiab] OR medline [tiab] OR pubmed [tiab] OR embase [tiab] OR Cochrane [tiab] OR scopus [tiab] or web of science [tiab] OR sources of information [tiab] OR data sources [tiab] OR following databases [tiab]) AND (study selection [tiab] OR selection criteria [tiab] OR eligibility criteria [tiab] OR inclusion criteria [tiab] OR exclusion criteria [tiab]) NOT #2 NOT #1 NOT ((systematic* [ti] AND review [ti]) or systemic review* [ti] or meta-analysis [ti] or metaanalysis [ti] or meta-analyses [ti] or metaanalyses [ti] or scoping review [ti] or scoping literature review [ti] or umbrella review [ti] or meta-synthesis [ti] or integrative review [ti])	17,057	13,424	78.7%

### Classification criteria

The criteria used for classifying articles as systematic reviews were based on those utilized in other studies [4, 10, 12, 17, 18, 45] and on the structure of Cochrane systematic reviews:

- The title or abstract describes the article as a systematic review.
- The methods section indicates that a systematic literature search was performed, specifies the databases searched, and presents a search strategy or search terms.
- The criteria for the inclusion/exclusion of a study are specified.
- A PRISMA flow diagram illustrating the process for selecting studies for analysis is presented.
- A meta-analysis is conducted for data synthesis or a qualitative synthesis of the evidence from the studies included in the review is available.
- The article presents a structured abstract (explicit or implicit) with the following sections: information sources/databases/search methods; criteria for study selection; data extraction; synthesis of data or evidence; and conclusions. Alternatively, the full text of the article reports information sources, search strategy or search terms, inclusion/exclusion criteria, and data extraction method.

An article was classified as systematic review only if one of the first two criteria (a or b) and one of the last four criteria (c, d, e, or f) were met. Reviews were excluded if the study selection criteria or conclusions were based on authors' personal and subjective experiences.

Three researchers independently reviewed the titles and abstracts of the each retrieved article. When the abstracts were not sufficiently informative, the full text was also examined. Disagreements among researchers were resolved through discussion and consensus.

### Identification of the reference set

The reference set is typically the best test currently available and the standard for comparisons [44]. To compare the properties of our developed filter and fulfill the objective of this study, the set retrieved by the PubMed SR filter was chosen.

### Evaluation of search strategies

Search strategy performance was evaluated by two measures: 1) the number of articles retrieved that were systematic reviews but were not included in the reference set (i.e., “New SR”) and 2) precision. Given the difficulty of calculating recall for a database with nearly thirty-two million records as of November 2020 and an unknown total number of systematic reviews, New SR was calculated, which directly affects the recall of the reference set. Precision was defined as the proportion of retrieved articles that were potential systematic reviews (expressed as a percentage). Precision was measured by screening all articles retrieved by each search strategy, except when the number of articles retrieved was greater than 2,000. For these cases, a random sample with the following parameters was selected from each set of results: 95% confidence level, 3% precision, and p=q=0.5; random samples were generated using the SPSS v.22 software package.

### Development and evaluation of the final search filter

The final search filter consisted of both text words from the search strategies that achieved a high degree of precision and MeSH terms for indexing relevant publication types (i.e., “systematic review [pt],” “meta-analysis [pt]”). A value of 70% was established as a cut-off point as it represents a high level of precision and corresponds to the statistical classification of a good correlation coefficient (≥0.7).

Because the final test of a search filter involves checking how well it performs in the database and interface for which it was designed [46], filter performance was evaluated in the full PubMed database by comparing the total number of articles retrieved with this filter to those retrieved by the PubMed SR filter.

## RESULTS

### Validation of the search strategy with terms obtained from the abstracts of systematic reviews published in the *Cochrane Database of Systematic Reviews* journal

Table 1 shows the set formed by all articles published in *Cochrane Database of Systematic Reviews*. After excluding certain publication types and articles with no abstract, 14,459 articles were retrieved (set #4). Set #5 contained terms selected from abstracts, achieving a recall of 98.9% because only 157 articles were missed (set #6). Of these articles, 49 were reviews of reviews with the following phrases in the titles: “overview of Cochrane reviews,” “overview of Cochrane systematic reviews,” and “overview of systematic reviews.” Another 86 articles were review protocols identified as such in the abstract, 8 were withdrawn reviews, and 14 lacked identifying features. Therefore, the selected terms were excellent identifiers of Cochrane reviews.

The proposed terms performed better than those used in the PubMed SR filter for identifying Cochrane systematic reviews from article titles (set #7). The elements of the PubMed SR filter used in this test achieved very low recall because 14,440 articles did not contain these terms in the title field. Only 19 articles were retrieved (14,459 minus 14,440), representing a recall of 0.13%. Our search strategy also performed slightly better than the systematic review [pt] search statement incorporated into the PubMed SR filter that achieves a recall of 97.9%, which did not retrieve 304 articles.

### Evaluation of proposed search strategies

Table 3 presents the performance of all tested search strategies (18) grouped by conceptual categories. For each strategy, the number of articles retrieved is shown after excluding items from the subset of PubMed systematic reviews (set #1) and certain publication types (set #2). The table also shows the number of reviews classified as systematic and not identified as such in the reference set (contributing to the recall increase for New SR) and the precision of the articles retrieved. A random sample was used in sets #3, #7, #10, #16, #17, and #18.

Set #5 retrieved 86 new Cochrane reviews (31 not published in *Cochrane Database of Systematic Reviews* and 55 reviews of Cochrane reviews). The 383 remaining were Cochrane reviews published in both *Cochrane Database of Systematic Reviews* and other journals as well as summaries, synopses, and abridged reviews. The calculated precision was the result of dividing 649 retrieved articles between 469 systematic reviews.

The highest precision was achieved by the “umbrella review” (96.7%) and “scoping review” OR “scoping literature review” OR “mapping review” (96.5%) strategies.

The “systematic synthesis” strategy retrieved few articles, most of which concerned the synthesis of chemical substances. Consequently, this search strategy was not incorporated into the filter, as its precision is very low. The “systematic search” phrase, which only achieved a precision of 20.9%, was also not incorporated into the filter. The search strategies of sets #15 and #16 achieved low overall precision (47.5% and 33.0%, respectively), and none of their phrases obtained precision greater than 70% (Appendix 3).

### Final search filter and comparison with reference set

Table 4 shows a comparison of the performance of the final search filter with the PubMed SR filter. Set #3 contains the search terms (text words found in systematic reviews and meta-analyses as publication types) from the sets in Table 3 that achieved an individual precision greater than 72% (between 72.3% and 96.7%, with the weighted mean precision being 83.8%). A total of 257,989 articles were retrieved after the publication types indicated in set #2 were excluded, 103,374 of which were not identified by the PubMed SR filter (set #6).

**Table 4:** Final search filter for the identification of systematic reviews and comparison of its performance with the PubMed SR filter (Date of search: December 16, 2020)

Search	Query	Items	Notes
#1	systematic [sb]	174,434	PubMed SR filter
#2	LETTER [PT] OR EDITORIAL [PT] OR COMMENT [PT] OR CASE REPORTS [PT] OR HISTORICAL ARTICLE [PT] OR REPORT [TI] OR PROTOCOL [TI] OR PROTOCOLS [TI] OR WITHDRAWN [TI] OR RETRACTION OF PUBLICATION [PT] OR RETRACTION OF PUBLICATION AS TOPIC [MESH] OR RETRACTED PUBLICATION [PT] OR REPLY [TI] OR PUBLISHED ERRATUM [PT]	4,594,185	
#3	(systematic* [ti] AND review [ti]) OR Systematic overview* [ti] OR Cochrane review* [ti] OR systemic review* [ti] OR scoping review [ti] OR scoping literature review [ti] OR mapping review [ti] OR Umbrella review* [ti] OR (review of reviews [ti] OR overview of reviews [ti]) OR meta-review [ti] OR (integrative review [ti] OR integrated review [ti] OR integrative overview [ti] OR meta-synthesis [ti] OR metasynthesis [ti] OR quantitative review [ti] OR quantitative synthesis [ti] OR research synthesis [ti] OR meta-ethnography [ti]) OR Systematic literature search [ti] OR Systematic literature research [ti] OR meta-analyses [ti] OR metaanalyses [ti] OR metaanalysis [ti] OR meta-analysis [ti] OR meta-analytic review [ti] OR meta-analytical review [ti] OR meta-analysis [pt] OR ((search* [tiab] OR medline [tiab] OR pubmed [tiab] OR embase [tiab] OR Cochrane [tiab] OR scopus [tiab] or web of science [tiab] OR sources of information [tiab] OR data sources [tiab] OR following databases [tiab]) AND (study selection [tiab] OR selection criteria [tiab] OR eligibility criteria [tiab] OR inclusion criteria [tiab] OR exclusion criteria [tiab]))	278,289	New filter—Text words (search strategies in title and abstracts fields)
#4	#1 NOT #2	168,677	PubMed SR filter
#5	#3 NOT #2	257,989	New filter—Text words
#6	#5 NOT #4	**103,374**	Potential SR found by the text words of New Filter, and not by the PubMed SR filter
#7	#4 AND #5	**154,615**	
#8	#4 OR #5	**272,051**	Potential SR in PubMed database
#9	(#3 OR systematic review [pt]) NOT #2	**272,048**	New full filter
#10	Systematic review [pt] NOT #2	137,305	
#11	#10 NOT #5	**14,059**	SR indexed as [pt] not retrieved by the text words of new filter
#12	#4 NOT #10	**31,372**	Potential SR retrieved by PubMed SR filter not indexed as SR [pt]
#13	#5 NOT #10	**134,743**	Potential SR retrieved by the text words of new filter not indexed as SR [pt]

The search terms of the new filter retrieved 154,615 articles from the PubMed SR filter (set #7), which means that they achieved a recall with respect to the reference standard of 91.6% (154,615/168,677). The number of articles that were not retrieved is 14,059, all of which were indexed as systematic review [pt] except for three that were withdrawn reviews. To avoid missing these articles, we incorporated the “systematic review [pt]” term (set #9) into the final search filter, which retrieves all PubMed SR filter articles and those retrieved by the search terms selected from titles and abstracts of systematic reviews except the three withdrawn reviews (as can be verified in set #8). The final search filter is as follows:

((systematic* [ti] AND review [ti]) OR Systematic overview* [ti] OR Cochrane review* [ti] OR systemic review* [ti] OR scoping review [ti] OR scoping literature review [ti] OR mapping review [ti] OR Umbrella review* [ti] OR (review of reviews [ti] OR overview of reviews [ti]) OR meta-review [ti] OR (integrative review [ti] OR integrated review [ti] OR integrative overview [ti] OR meta-synthesis [ti] OR metasynthesis [ti] OR quantitative review [ti] OR quantitative synthesis [ti] OR research synthesis [ti] OR meta-ethnography [ti]) OR Systematic literature search [ti] OR

Systematic literature research [ti] OR meta-analyses [ti] OR metaanalyses [ti] OR metaanalysis [ti] OR meta-analysis [ti] OR meta-analytic review [ti] OR meta-analytical review [ti] OR meta-analysis [pt] OR ((search* [tiab] OR medline [tiab] OR pubmed [tiab] OR embase [tiab] OR Cochrane [tiab] OR scopus [tiab] or web of science [tiab] OR sources of information [tiab] OR data sources [tiab] OR following databases [tiab]) AND (study selection [tiab] OR selection criteria [tiab] OR eligibility criteria [tiab] OR inclusion criteria [tiab] OR exclusion criteria [tiab])) OR systematic review [pt])

NOT (letter [pt] OR editorial [pt] OR comment [pt] OR case reports [pt] OR historical article [pt] OR report [ti] OR protocol [ti] OR protocols [ti] OR withdrawn [ti] OR retraction of publication [pt] OR retraction of publication as topic [mesh] OR retracted publication [pt] OR reply [ti] OR published erratum [pt])

The new full search filter achieved a considerable increase in the number of retrieved articles that were potential systematic reviews (i.e., 61.3% more, 272,048/168,677), with a likely high degree of precision. The PubMed SR filter retrieved 62.0% (168,677/272,048) of the articles of our final filter, which means that it is likely to have missed a large number of potential systematic reviews.

A total of 18.6% of systematic reviews included in the PubMed SR filter were not indexed as “systematic review [pt]” (31,372/168,677), whereas text words of our filter retrieved 134,473 articles not indexed as “systematic review [pt]” (4.3-fold increase).

We also excluded not only the publication types listed in the PubMed filter (i.e., protocols and comments) but also those indicated in set #2. This process excluded 5,757 (174,434 minus 168,677) articles indexed as other publication types.

## DISCUSSION

The newly developed search filter consists of 1) search strategies with terms extracted from abstracts of reviews published in *Cochrane Database of Systematic Reviews* and the titles of articles indexed as “systematic review” [pt] and 2) MeSH terms for indexing systematic review or meta-analysis as a publication type. This approach enabled us to create a search filter to identify both Cochrane and non-Cochrane systematic reviews, improving the performance of the PubMed SR filter by substantially increasing the number of retrieved potential systematic reviews with an average precision of potentially 83.8% within this sample.

Various factors such as creation dates, databases, and platforms used as well as the article sets created for evaluation and/or validation of the results limit our ability to compare the results of our filter with those achieved in other filter studies. Hence, the following paragraphs present an analysis and assessment of the terms and syntax used and their effects on the retrieval and precision of the results.

Appendix 4 shows a table with the terms used in our search filter and in the strategies of other studies [10–13, 16, 18, 31, 45], as well as those used by the BMJ Knowledge Center [47], Canadian Agency for Drugs and Technologies in Health [48], and PubMed SR filter [33]. Other studies featuring strategies that only use and/or contain the terms “meta-analysis” and/or “systematic review” were disregarded [4, 14, 17].

### Search terms identifying systematic reviews

Terms usually used to describe studies that perform a rigorous, replicable, and structured synthesis of research are “systematic review,” “systematic overview” [27, 38], and “Cochrane review.”

The search strategies of all the discussed articles contain the phrase “systematic review,” although the syntax used varies. Two fundamental aspects of the search process should be noted. First, given that many phrases contain at least one word between the two terms (Appendix 5), using a proximity operator such as ADJn in Ovid is recommended to retrieve all possible combinations. Since PubMed does not support proximity operators, the AND operator must be used to combine the two terms, as searching for them as a phrase excludes a considerable number of potentially relevant articles. Second, the results will be more precise if the search targets the title field instead of text fields (i.e., title and abstract). Our strategy matches the strategies used by Shojania and Bero [16] and Lunny et al. [10] in that the two terms combined with AND are searched in the title field.

Other systematic approaches for synthesizing evidence are scoping reviews (also called mapping reviews [49]), umbrella reviews, and integrative reviews [50]. The scoping reviews are useful for both emerging and established research topics [51, 52] and feature many key components of systematic reviews [53], such as the search, analysis, and synthesis of literature, and are a valuable input for future systematic reviews [54]. None of the studies analyzed used the terms “scoping” or “mapping” in their filter. Only the PubMed SR filter includes these two terms but as phrases accompanied by the term systematic (“systematic scoping review [ti]” or “systematic mapping review [ti]”), resulting in the lack of retrieval of a large number of articles that only contain “scoping review” or “mapping review” in the title field.

The recent growth in the number of systematic reviews published has resulted in the creation of reviews synthesizing evidence from multiple systematic reviews. Various terms have been used to describe this type of review, such as “overview of reviews” [55], “review of reviews,” “meta-review” [56], and “umbrella review” [57]. Only Lunny [10] uses these terms, and PubMed includes the phrase “systematic meta-review” but does not retrieve all meta-reviews not accompanied by the word “systematic.”

Integrative reviews combine studies with different methods to improve the understanding of a topic [58]; they are included only in the BMJ Knowledge Center [47] and CADTH [48] search strategies.

We also used the phrase “systemic review” in our search strategy. We did not find this phrase in any of the filters analyzed and published in literature, although it increases recall more than the previously mentioned terms with an individual precision of 89%. “Systemic” has a different meaning as “systematic,” so the only plausible explanation is that the term “systematic” was misused, incorrectly translated, or a typo.

Other terms are also used to indicate a synthesis of evidence or research, such as “research synthesis,” which is defined as an integration of empirical research for the purpose of making generalizations [59]. Research syntheses can be either quantitative or qualitative. Reviews synthesizing the results of qualitative studies are called meta-syntheses [60] or qualitative research syntheses. This category includes meta-ethnography reviews [61], which synthesize results based on a systematic literature review [62]. None of the studies discussed here uses these terms.

Other candidate terms for retrieving reports of potential systematic reviews are “quantitative reviews” and “quantitative synthesis,” which are used in more than half of the studies discussed. These terms represent concepts and reflect the evolution of the evidence-based movement over the years and its extension and application to other areas (e.g., public health, service delivery) and different areas of medicine.

Some systematic reviews include meta-analyses, but not all meta-analyses are systematic reviews, as shown by the individual precision of our search strategy (84.8%). Therefore, the term “meta-analysis” must be used in any search strategy designed to retrieve systematic reviews. In fact, it appears in all discussed studies. Surprisingly, it is not included in the PubMed SR filter, thus explaining its failure to retrieve more than 64,000 articles in the present study. These terms can have different spellings: with or without a hyphen and ending in “is” or “es” (i.e., meta-analysis, metaanalysis, meta-analyses, meta-analyses). Therefore, all variations must be either included in the search strategy or truncated, as done by Boynton et al. [12] and Hunt and McKibbon [31]. In addition, meta-analysis must be searched as publication type (i.e., meta-analysis [pt]) and always complemented by searching in the title field, as not all meta-analyses are indexed by publication type and the term is not always correctly tagged [12].

Although very few articles with the phrase “systematic literature search” or “systematic literature research” were retrieved, the achieved high levels of precision support the inclusion of these phrases in the strategy. Only Bramer et al. use these in their filter [45].

The final part of our search strategy addresses two essential concepts that, according to the PRISMA Statement [35], should be included in both the abstract and methods sections of a systematic review: search strategy, information sources or databases, and inclusion/exclusion criteria for study selection.

In all but one study, terms referring to specific databases (e.g., MEDLINE, EMBASE, PubMed) are included in the search strategy. These terms are sometimes individually searched and other times combined with review articles (i.e., AND review [pt]) [31] or with searches (i.e., AND search* [tw]) [11]. We also added the concept of eligibility criteria to the search strategy to improve its precision, as we believe that only including terms referring to databases may not distinguish a systematic review from narrative reviews. This strategy retrieved a large number (13,424) of systematic reviews that were not found by the PubMed SR filter (Table 3, set #18), achieving relatively high precision (78.7%).

Despite inclusion of the “systematic review [pt]” MeSH term in 2019 in the PubMed SR filter, up to 31,372 potential systematic reviews not indexed by publication type were retrieved by the PubMed SR filter, which increased to 134,743 with our strategy (Table 4, set #13). This is a very large number of articles that requires searching for single terms or phrases in topic fields which identify systematic reviews to avoid missing relevant articles not indexed or incorrectly indexed with this MeSH term.

We also decided not to include very generic terms used in some studies, which undoubtedly achieve very high recall values but have low individual precision (e.g., review [pt], review [tiab], published adj studies.ab, data adj extraction.ab, summary [ti] AND articles [ti], analysis [ti] AND articles [ti], intervention$.ti.). Instead, we used highly specific terms in our final filter to increase the number of potential systematic reviews retrieved (i.e., recall) and to achieve a high degree of precision.

### Search strategy syntax

Two aspects of syntax affect search recall and precision: 1) how to search for phrases with two or more terms and 2) where to search (i.e., fields).

One of the most effective ways to search for phrases in PubMed is adding a field label at the end of the phrase, thus retrieving articles that contain adjacent terms that are in the same order. Our search filter uses this approach in all cases except when searching for “systematic reviews.” In this case, the terms are combined with the AND operator. As previously explained, the reason is that many phrases include at least one term between “systematic” and “review.” Consequently, it is necessary to search in this manner to avoid missing studies and hence reducing recall. The risk of this type of search is lower precision, although limiting the search to the title field will reduce the noise level.

Regarding the search fields, except when a particular term is searched as a publication type (e.g., review [pt], meta-analysis [pt]) or as a MeSH term (e.g., meta-analysis [mesh], medline [mesh]), the strategies of all discussed studies search in the title field [ti], in the title and abstract fields [tiab], or in text words [tw] fields. The search method used is determined by the desired precision. Searching in the title field provides more precise results than in the title and abstract fields.

Strategies with low precision can consume excessive time and resources for examining and reading the retrieved articles. Our search filter mostly searches in the title field (i.e., [ti]) to achieve as high precision as possible and to optimize the time spent identifying systematic reviews. Only terms extracted from the abstracts of Cochrane reviews were searched in the title and abstract (i.e., [tiab]) fields.

The PubMed SR filter searches for different types of systematic reviews in the title field (e.g., systematic review, systematic Cochrane review, systematic narrative review, systematic qualitative review, systematic scoping review, systematic mapping review, systematic meta-review, systematic quantitative review). Our filter covers all these terms by combining “systematic” and “review.” However, including the term “systematic” in the PubMed SR filter requires the terms to be searched as a phrase, resulting in a likely lower recall of potentially relevant articles because review types that do not contain the term “systematic” in the title field (e.g., scoping review, quantitative review) are not retrieved.

The proposed filter searches for terms in the title and abstract fields that should appear in the abstract of a systematic review according to the PRISMA Statement [35]. Recently, PRISMA-S [63] was published as a guide to help authors more clearly and reproducibly record the search methods and names of the most important databases. Therefore, author compliance with PRISMA guidelines in the future is likely to improve the performance of this filter and increase the likelihood of correct indexing of systematic reviews as a publication type.

### Limitations

The main limitation of this study is the broad definition of systematic review and the criteria used to classify studies as such, as these criteria do not include a quality assessment of the selected articles. For some authors, a systematic search and screening to identify evidence does not necessarily mean that a study is a systematic review [64]. Therefore, we recognize that although a review may have followed a systematic methodology and explicitly indicated the use of a search strategy, it is not necessarily a systematic review in the strictest sense. However, the same observation can be applied to publication types included in the PubMed SR filter (e.g., literature review, narrative review) or other published reviews, even though their authors describe them as systematic [65]. Other issues hindering the correct identification of this publication type and its classification as systematic review are lack of transparency, poor methodological quality, and incomplete documentation of various PRISMA Statement requirements [23, 66–70]. Nevertheless, we believe that the criteria used in this study filter out other types of nonsystematic reviews, such as narrative reviews.

Another limitation of our study is that an unknown number of systematic reviews may have been overlooked due to the variety of terms and synonyms used in literature to describe reviews with systematic approaches.

Although the developed filter retrieved more potential systematic reviews than the PubMed SR filter with a high degree of precision, it was not validated using a gold standard article set or real case studies; therefore, the actual recall and its performance in these cases is unknown.

### Conclusions

The developed search filter provides higher recall than the PubMed SR filter for retrieving potential systematic reviews with a likely high degree of precision. The practical application of this search filter has two useful implications:

- It can assist doctors, researchers, librarians, and information professionals in efficiently searching and locating a larger number of potential systematic reviews than the PubMed SR filter. This could help them to reach better-informed decisions in their clinical practices and conduct less-biased studies of reviews.
- It will allow searchers and researchers to find more articles. Although this will require more time for screening, the number needed to read is likely to be low, so searchers' time, although lengthened, will feel usefully spent. In addition, combining this strategy with specific topic terms will further increase precision.

Finally, given the evolution of the evidence-based movement and the growth of systematic approaches to literature reviews, including possible changes over time in the use of terminology, this and other search filters for systematic reviews should be updated frequently.

## Data Availability

Data associated with this article are available at https://osf.io/6mntu/?view_only=dbbdea8d7632488391ab941d1fe7ffbf.

## SUPPLEMENTAL FILES

**Appendix 1.** Terms and frequency

**Appendix 2.** Concepts and terms

**Appendix 3.** Tables 5 and 6

**Appendix 4.** Comparison of the terms used in the final search filter with those used in other studies

**Appendix 5.** Phrases of articles that contain the terms systematic and review in the title and are not retrieved in the systematic reviews [sb]
