# Supplementary material for: Development of an efficient search filter to retrieve systematic reviews from PubMed
Source: J Med Libr Assoc. 2021 Oct 1;109(4):561–74. doi: 10.5195/jmla.2021.1223 (PMC8608217; doi:10.5195/jmla.2021.1223)
Supplement: Supplementary file 3 — Appendix 3. Tables 5 and 6 [file jmla-109-4-561-s03.docx]

Appendix 3.

Table 5. Precision of terms related with systematic appraisal of literature or evidence (set 15 of table 3)

|  | Non systematic reviews | Systematic reviews | Total Items |
| --- | --- | --- | --- |
| Systematic analysis AND evidence | 10 33.3% | 20 66.7% | 30 |
| Systematic analysis AND literature | 13 34.2% | 25 65.8% | 38 |
| Systematic analysis AND research | 19 67.9% | 9 32.1% | 28 |
| Systematic analysis AND studies | 21 72.4% | 8 27.6% | 29 |
| Systematic analysis AND trials | 9 75.0% | 3 25.0% | 12 |
| Systematic appraisal AND evidence | 1 33.3% | 2 66.7% | 3 |
| Systematic appraisal AND literature | 2 50.0% | 2 50.0% | 4 |
| Systematic appraisal AND research | 1 100.0% | 0 0.0% | 1 |
| Systematic appraisal AND studies | 1 100.0% | 0 0.0% | 1 |
| Systematic appraisal AND trials | 2 40.0% | 3 60.0% | 5 |
| Systematic assessment AND evidence | 3 50.0% | 3 50.0% | 5 |
| Systematic assessment AND literature | 1 50.0% | 1 50.0% | 3 |
| Systematic assessment AND research | 3 60.0% | 2 40.0% | 5 |
| Systematic assessment AND studies | 3 42.9% | 4 57.1% | 7 |
| Systematic assessment AND trials | 4 66.7% | 2 33.3% | 6 |
| Systematic evaluation AND evidence | 4 36.4% | 7 63.6% | 11 |
| Systematic evaluation AND literature | 4 44.4% | 5 55.6% | 9 |
| Systematic evaluation AND research | 9 75.0% | 3 25.0% | 12 |
| Systematic evaluation AND studies | 6 46.2% | 7 53.8% | 13 |
| Systematic evaluation AND trials | 8 57.1% | 6 42.9% | 14 |
| TOTAL | 124 52.5% | 112 47.5% | 236 |

Table 6. Precision of terms related with evidence (random sample of the items retrieved from set 16 of table 3)

|  | Non systematic reviews | Systematic reviews | Total Items |
| --- | --- | --- | --- |
| Review AND evidence | 235 56.4% | 182 43.6% | 417 |
| Synthesis AND evidence | 157 87.2% | 23 12.8% | 180 |
| Evidence-based review | 30 32.6% | 62 67.4% | 92 |
| Evidence-based approach | 72 83.7% | 14 16.3% | 86 |
| Scientific evidence | 47 88.7% | 6 11.3% | 53 |
| Update AND evidence | 28 60.9% | 18 39.1% | 46 |
| Evidence-based treatment | 26 86.7% | 4 13.3% | 30 |
| Evidence-based management | 19 86.4% | 3 13.6% | 22 |
| Overview AND evidence | 18 81.8% | 4 18.2% | 22 |
| Evidence-based recommendations | 14 70.0% | 6 30.0% | 20 |
| Critical appraisal AND evidence | 12 80.0% | 3 20.0% | 15 |
| Critical evaluation AND evidence | 3 75.0% | 1 25.0% | 4 |
| Evidence-based overview | 3 75.0% | 1 25.0% | 4 |
| TOTAL | 664 67.0% | 327 33.0% | 991 |
