# Supplementary material for: Development of an efficient search filter to retrieve systematic reviews from PubMed
Source: J Med Libr Assoc. 2021 Oct 1;109(4):561–74. doi: 10.5195/jmla.2021.1223 (PMC8608217; doi:10.5195/jmla.2021.1223)
Supplement: Supplementary file 4 — Appendix 4. Comparison of the terms used in the final search filter with those used in other studies [file jmla-109-4-561-s04.docx]

Appendix 4. Comparison of the terms used in the final search filter with those used in other studies.

|  | **Shojania^16^** | **Wong^11^** | **Boynton^12^** | **Lee^13^** | **Montori^18^** | **Lunny^10^** | **Hunt^31^** | **Bramer^45^** | **BMJ Knowledge Center^47^** | **CADTH^48^** | **US NLM^33^** |
| --- | --- | --- | --- | --- | --- | --- | --- | --- | --- | --- | --- |
| Systematic [ti] and review* [ti] | Systematic [ti] and review [ti] | Systematic review.tw | Systematic$ adj4 review$.tw | Systematic review.tw | Systematic [tiab] and review [tiab] | Systematic [ti] and reviews [ti] | Systematic review.tw | Systematic review [tiab]  Systematically review* [tiab]  Systematic literature review* [tiab] | Systematic adj5 review*.tw | Systematic review*[tiab] | Systematic review [ti] |
| Systematic overview* [ti] | Systematic [ti] and overview [ti] |  | Systematic$ adj4 overview$.tw |  |  | Systematic [ti] and overview* [ti] | Systematic overview*.tw |  | Systematic adj5 overview*.tw | Systematic overview*[tiab] |  |
| Cochrane review* [ti] | Cochrane [tw] and review [ti] |  |  |  |  | Cochrane [ti] and reviews [tiab]  Cochrane [ti] and overview* [ti] |  |  |  |  | Systematic Cochrane review [ti] |
| Systemic review* [ti] |  |  |  |  |  |  |  |  |  |  |  |
| Scoping review [ti] OR scoping literature review [ti] OR mapping review [ti] |  |  |  |  |  |  |  |  |  |  | Systematic scoping review [ti]  Systematic mapping review [ti] |
| Umbrella review [ti] |  |  |  |  |  | Umbrella review [tiab] |  |  |  |  |  |
| Review of reviews [ti] OR overview of reviews [ti] OR meta-review [ti] |  |  |  |  |  | Review[ti] and reviews [ti]  Overview[ti] and reviews [ti]  Meta-review [tiab] or metareview [tiab] |  |  |  |  | Systematic meta-review [ti] |
| meta-synthesis [ti] OR metasynthesis [ti] OR meta-ethnography [ti] |  |  |  |  |  |  |  |  |  |  |  |
| integrative review [ti] OR integrated review [ti] OR integrative overview [ti] |  |  |  |  |  |  |  |  | integrative research review$.tw | Integrative review*[tiab]  Integrative overview[tiab] |  |
| quantitative review [ti] OR quantitative synthesis [ti] OR research synthesis [ti]) | Quantitative [ti] and synthesis [ti] |  | Data adj synthesis.tw |  |  |  | Quantitative review* [tw]  Quantitative overview* [tw] |  | Quantitativ$ adj5 review$.tw  Quantitativ$ adj5 overview$.tw  Quantitativ$ adj5 synthesis$.tw | Quantitative review*[tiab]  Quantitative overview*[tiab]  Quantitative synthes*[tiab] | Systematic quantitative review [ti] |
| systematic literature search [ti] OR  systematic literature research [ti] | Literature [ti] and (synthesis [ti] or overview [ti] or review [ti]) |  |  |  |  |  |  | Systematic search* [tiab]  Systematically search* [tiab]  Systematic literature search [tiab] |  |  | Systematic literature review [ti] |
| meta-analyses [ti] OR metaanalyses [ti] OR metaanalysis [ti] OR meta-analysis [ti] OR meta-analytic review [ti] OR meta-analytical review [ti] OR meta-analysis[pt] | Meta-analysis [pt]  Meta-analysis [tw]  Metanalysis [tw] | Metaanalysis.mp.pt | Meta-analysis.sh  Meta-analy$.tw  Metaanal$.tw  Meta-analysis.pt | Meta-analysis.pt  Meta-analysis [tiab] | Meta-analysis [pt]  Meta-analysis [tiab]  Metaanalysis [mesh] | Meta-analyses [ti] | Meta-analysis [pt]  Metaanal* [tw]  Meta-anal* [tw] |  | Meta-analysis.pt  Meta-analys$.tw  Meta analys$.tw  Metaanalys$.tw | Meta-analysis[pt]  Meta-analysis[mh]  Meta-analy*[tw]  Metanaly*[tw]  Metaanaly*[tw] |  |
| (search* [tiab] OR medline [tiab] OR pubmed [tiab] OR embase [tiab] OR Cochrane [tiab] OR scopus [tiab] or web of science [tiab] OR sources of information [tiab] OR data sources [tiab] OR following databases [tiab]) AND (study selection [tiab] OR selection criteria [tiab] OR eligibility criteria [tiab] OR inclusion criteria [tiab] OR exclusion criteria [tiab]) | Search* [tw] and (Cochrane [tw] OR Medline [tw] OR CINAHL [tw] OR database* [tw]) | Search.tw  Medline.tw | Search.ab  Medline.ab | Medline.tw | Search [tiab] or  Medline [tiab] |  | Review [pt] and medline [tw] | Medline [tiab]  Embase [tiab]  Pubmed [tiab]  Prisma [tiab]  Google scholar [tiab] | (MEDLINE or embase or pubmed or cochrane).tw,sh  electronic database$ or bibliographic database$ or computeri?ed database$ or online database$).tw,sh | Embase[tiab]  Cochrane[tiab]  Medline[tiab]  Pubmed[tiab] |  |
| Systematic review [pt] |  |  |  |  |  |  |  |  |  | Systematic [sb] | Systematic review [pt] |
