# Supplementary material for: Development of an efficient search filter to retrieve systematic reviews from PubMed
Source: J Med Libr Assoc. 2021 Oct 1;109(4):561–74. doi: 10.5195/jmla.2021.1223 (PMC8608217; doi:10.5195/jmla.2021.1223)
Supplement: Supplementary file 5 — Appendix 5. Phrases of articles that contain the terms systematic and review in the title and are not retrieved in the systematic reviews [sb] [file jmla-109-4-561-s05.docx]

Appendix 5. Phrases of articles that contain the terms systematic and review in the title and are not retrieved in the systematic reviews [sb]

| systematic analytical review |
| --- |
| systematic and clinical review |
| systematic and comparative review |
| systematic and comprehensive review |
| systematic and critical mixed-methods review |
| systematic and critical review |
| systematic and descriptive review |
| systematic and integrative review |
| systematic and literature review |
| systematic and meta-analytic review |
| systematic and meta-analytical review |
| systematic and meta-review |
| systematic and methodological review |
| systematic and narrative review |
| systematic and psychometric review |
| systematic and qualitative review |
| systematic and quality review |
| systematic and quantitative review |
| systematic and realistic reviews |
| systematic and review analysis |
| systematic bibliographic review |
| systematic case review |
| systematic clinical data review |
| systematic clinical review |
| systematic comparative effectiveness review |
| systematic comprehensive literature review |
| systematic critical literature review |
| systematic data review |
| systematic descriptive review |
| systematic economic review |
| systematic epidemiological review |
| systematic evidence based practice review |
| systematic evidence based review |
| systematic experiment literatures review |
| systematic global review |
| systematic grade-based review |
| systematic guideline review |
| systematic health equity review |
| systematic huge review |
| Systematic integrated review |
| systematic integrative literature review |
| systematic literature analysis and review |
| systematic literature overview |
| systematic literature search and descriptive review |
| systematic literature search and narrative review |
| systematic map review |
| systematic meta-analytic review |
| systematic meta-ethnographic review |
| systematic meta-literature review |
| systematic meta-narrative review |
| systematic methodological review |
| systematic minireview |
| systematic mixed methods review |
| systematic multidisciplinary review |
| systematic narrative literature review |
| systematic narrative synthesis review |
| systematic neuroanatomical-based review |
| systematic neuropathological review |
| systematic neurophysiological review |
| systematic overview |
| systematic pathology review |
| systematic pharmacoepidemiological review |
| systematic pharmacology review |
| systematic policy review |
| systematic post-quantec review |
| systematic prisma review |
| systematic psychometric review |
| systematic pubmed review |
| systematic qualitative literature review |
| systematic qualitative meta-agregation review |
| systematic quality review |
| systematic quantitative literature review |
| systematic rapid review |
| systematic realist literature review |
| systematic realist review |
| systematic research review |
| systematic sampling review |
| systematic scoping literature review |
| systematic search and analytical review |
| systematic search and critical review |
| systematic search and literature review |
| systematic search and mapping review |
| systematic search and narrative review |
| systematic search and qualitative review |
| systematic search and rapid review |
| systematic semi-quantitative review |
| systematic shortcut review |
| systematic state-of-the-art review |
| systematic structured review |
| systematic study and review |
| systematic synthesis and narrative review |
| systematic synthesis and review |
| systematic technical review |
| systematic technology review |
| systematic thematic review |
| systematic translational review |
| systematic umbrella review |
| systematic update review |
| systematic worldwide review |
| systematic xx-year review |
| systematic/meta-analytic review |
| systematic, blinded review |
| systematic, criteria-based review |
| systematic, methodology-focused review |
| systematic, mixed studies literature review |
| systematic, multi-domain review |
| systematical literature review |
| systematical review of the literature |
| systematically conducted literature review |
| systematically conducted narrative review |
| systematized review |
| systemic literature review |
| systemic review |
| comprehensive review and approach systematic |
| comprehensive review with systematic analysis of the published data |
| critical review using systematic methods |
| review and systematic aanalysis |
| review based on systematic survey of the literature |
| review of qualitative studies using systematic methods |
| review of reviews and systematic update |
| review the systematic analysis |
| review using a systematic approach |
| review utilizing a systematic approach |
| review with a systematic approach |
| review with a systematic search |
